# Impact of Fish Farming on Phosphorus in Reservoir Sediments

**DOI:** 10.1038/srep16617

**Published:** 2015-11-18

**Authors:** Binyang Jia, Ya Tang, Liyan Tian, Leander Franz, Christine Alewell, Jen-How Huang

**Affiliations:** 1 Chengdu Academy of Environmental Sciences, Chengdu 610072, People’s Republic China; 2 College of Architecture and Environment, Sichuan University, Chengdu 610065, People’s Republic China; 3 Environmental Geosciences, University of Basel, CH-4056 Basel, Switzerland; 4 Mineralogy and Petrography, University of Basel, CH-4056 Basel, Switzerland

## Abstract

Fish farming has seriously influenced the aquatic environment in Sancha reservoir in SW China since 1985 and has been strongly restricted since 2005. Thus, phosphorus speciation in a sediment core dated between 1945 and 2010 at cm-resolution and in surface sediments from Sancha reservoir may allow us track how fish farming impacts phosphorus dynamics in lake sediments. Fish farming shifts the major binding forms of phosphorus in sediments from organic to residual phosphorus, which mostly originated from fish feed. Sorption to metal oxides and association with organic matters are important mechanisms for phosphorus immobilisation with low fish farming activities, whereas calcium-bound phosphorous had an essential contribution to sediment phosphorus increases under intensive fish framing. Notwithstanding the shifting, the aforementioned phosphorus fractions are usually inert in the lake environment, therefore changing phosphorus mobility little. The use of fish feed and water-purification reagents, the most important additives for fish farming, introduce not only phosphorus but also large amounts of sand-sized minerals such as quartz into the lake, to which phosphorus weakly sorbs. The sand-sized minerals as additional sorbents increase the pool of easily mobilisable phosphorus in sediments, which will slow down the recovery of reservoir water due to its rapid re-mobilisation.

Phosphorus (P) is an element that is essential for life and a key limiting nutrient in freshwater systems^1^. However, excessive amounts of P entering lakes from rivers and through a variety of human activities (e.g. aquaculture, industry and municipal sewage treatment^2^,^3^,^4^) lead to eutrophication and algal blooms in lakes^5^,^6^. The lake sediment may either act as a sink or an internal source for the overlying water, depending on the quantity of P discharged, environmental conditions and chemical forms of sediment-bound P^7^,^8^,^9^,^10^. To date, plenty of studies have investigated P dynamics in lakes of different trophic states under the influence of human activities, mostly the distribution and speciation of P in the waters and sediments^11^,^12^. However, detailed knowledge about the mechanisms responsible for human activities, especially fish farming in cage systems, changing the biogeochemical behaviour of P in the lake sediment is still scarce. Fish farming in cages is considered to be an intensive or semi-intensive farming technique. The production process releases a large amount of residual food and excreta into the water, which can be oxidised to soluble substances and partially consumed by lake planktons^13^,^14^. The majority of these by-products would become part of the sediments, ultimately leading to long-term accumulation^15^,^16^,^17^. Several studies have focused on the input and output budget of nutrient compounds e.g. C, N and P in fish farms, showing that more than 80% of the original P input is released from fish cage farms and lost in the marine or lake environment^10^,^16^,^17^,^18^. Fish farming increases not only P sedimentation but also changes P speciation e.g., accumulation of Ca-bound P (HCl reactive P)^19^,^20^ and biological activities in sediments e.g., an increment of alkaline phosphatase activity in lake sediments^21^. Still, the current understanding about the influence of fish farming in cages on P mobility and speciation in the sediments is very limited.

Sancha reservoir in Sichuan Province, Southwest China (Fig. 1), is impacted by a serious problem of eutrophication^15^,^22^. The major human activity prior to fish farming was terrestrial agriculture onshore and the aquatic environment in the reservoir was oligotrophic until 1985. The aquatic environment of Sancha reservoir turned intensely eutrophic with the introduction of fish farming in 1985 and other industrial activities from 2000 onwards. Although there were several measures by the local government to reduce anthropogenic P input drastically for the remediation of Sancha reservoir since 2005, the aquatic environment is still eutrophic today. The corresponding total P discharge into the lake increased from 5 t yr^−1^ in 1978 to 65 t yr^−1^ in 2005 and decreased to 23 t yr ^−1^ in 2010, while the P contents in the sediments ranged widely from 0.63 to 16.8 mg g^−1^ (Fig. 2a)^22^. Fish farming in cages in Sancha reservoir expanded from the initial cage area of 1,000 m^2^ in the late 1980s to a peak area of 255,300 m^2^ in 2005 but was largely reduced in 2010. In the process of cultivating fish, at least 10% of fish feed was estimated to deposit directly at the bottom of the Sancha reservoir, leading to an accelerated sedimentation rate of bottom sediments (see Figure S2)^22^. The large variations of fish farming activities in the history in Sancha reservoir allow us quantify the influence of fish farming on the vertical and spatial distribution, speciation and mobility of P in the lake sediments, which is the objective of this study. Global aquaculture production has grown at an average rate of 8.8% annually since 1980 and is expected to further increase in the future^23^. Therefore, our findings will provide scientific knowledge to improve and restore the reservoir’s ecological environment affected by fish farming.

## Results

### Phosphorus vertical distribution in the sediment profile

The general low geological background P content in Sancha reservoir allows a clear separation of natural and anthropogenic environmental events in the lake basin sediments (Fig. 2). The total sediment P contents prior to 1985 were constantly low (<2 mg g^−1^ beneath 27 cm in depth), which implied a non- to weak human activity in the Sancha reservoir region before 1985 (Fig. 2c). The P input prior to 1985 was predominately natural, inclusive of atmospheric deposition, surface runoff and discharge from the south canal of the Dujiangyan irrigation network. Domestic discharges accounted for ~30% of the total input and the contribution from fish farming was negligible (Fig. 2c). The content of sediment P in the layers dating from 1990 to 2005 increased rapidly as a result of drastic increases in anthropogenic P discharges into the lake. Most apparently, the sediment P content and anthropogenic P input peaked in both 1996 and 2005 (10 and 16.9 mg g^−1^ at 22 and 12 cm depth, respectively). The strong impact of human activities on P pollution in Sancha reservoir was also supported by the comparably low natural P input from the south canal of the Dujiangyan irrigation network discharge and from atmospheric deposition. In 1996 and 2005, the P discharge into the reservoir from the south canal of the Dujiangyan irrigation network and atmospheric P deposition were 10–15% and 1–2% of the total P input, respectively (Fig. 2c). The sediment P content declined gradually to 1.7 mg g^−1^ in the sediment layer younger than 2005, when the government started measures to reduce anthropogenic P discharge into Sancha reservoir. Among the origin of total P discharges into Sancha reservoir, the proportion of fish farming increased from 18% in 1990 to 81% in 2005 and then decreased to 43% in 2010. The corresponding absolute amount of P discharged from fish farming amounted to 2, 52.6, 10.1 tons, respectively. The sediment P was strongly and significantly correlated with the grain size distribution (the contents of P correlated positively with the proportion of sand-sized grain (r = 0.84, p < 0.001) but negatively with silt- and clay-sized grains (r = −0.86, p < 0.001; see Figure S4). This might be counterintuitive because fine-grained particles have a higher sorption capacity, and can only be explained by the concomitant input of P with sand-sized material. Sand (>64 μm) predominated in the same sediment layer where P contents peaked, whereas there was almost no detectable sand sized particles below 25 cm^22^ (Figure S3).

### Phosphorus fractionation in the sediment profile

The sediment layers could be categorised into three groups based on the distinctly different correlations of different P fractions with total P and other elements (Fig. 3 and S5). Significant correlations will help to identify the P fractions ruling the fluctuation of total P contents in sediments and reveal the elements involved in P sedimentation. The first group included the sediment layers from 42 to 27 cm depth (dated between 1945 and 1985) in which the activity of fish farming was still negligible with almost constant P contents (< ~1 mg g^−1^) and P species distribution (Fig. 2b). Organic P and HCl-P were the major components with comparably constant percentages, accounting together for >50% of total P, while in the younger sediments they were all below 50% (Fig. 2b). A significant correlation was only found between total P and organic P contents (r = 0.96, p < 0.001, Fig. 3e). The sediment layers from 26 cm depth to the water-sediment interface (dated younger than 1987), in which residual P predominated (~30–50%), can be further separated into two groups, taking 3.5 mg g^−1^ of total P in the sediment as the threshold content for the changes of correlative relationship (Fig. 3 and S5). At low P levels (total P < 3.5 mg g^−1^), total P correlated positively with MgCl_2_-P (r = 0.62, p = 0.018, Fig. 3a), NaOH-P (r = 0.63, p = 0.016, Fig. 3c), and organic P (r = 0.73, p = 0.003, Fig. 3e). Moreover, organic P correlated positively (r = 0.91, p < 0.001) with C contents (Figure S5). In comparison, there were highly significant and positive correlations between total P and MgCl_2_-P (r = 0.92, p < 0.001, Fig. 3a) and HCl-P (r = 0.91, p < 0.001, Fig. 3d) at high P levels (total P > 3.5 mg g^−1^). An additional correlation was found between HCl-P and Ca contents (r = 0.83, p < 0.001, Figure S5). Residual P was the only fraction which correlated significantly with total P at both low and high P levels and the dependence was almost identical (r = 0.94 and 0.96, respectively, p < 0.001, Fig. 3f).

### Spatial distribution and characterisation of phosphorus in surficial sediments

**Figure 1:**
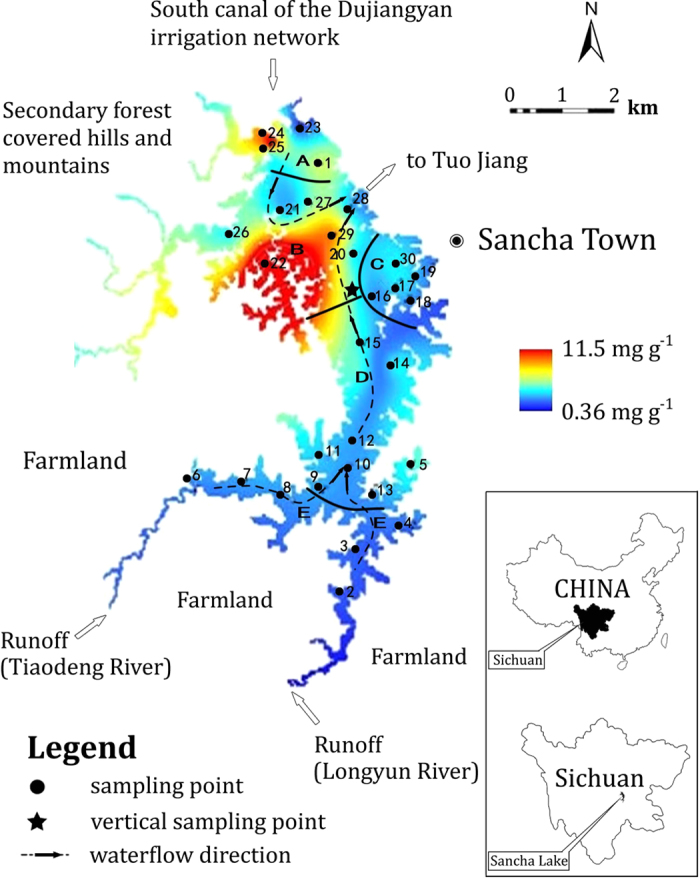
Location of Sancha Lake in Sichuan Province, China. (**A–E**) represent the sampling districts. (**A**) the main reservoir inflow (south main canal) zone, (**B**) the highly concentrated fish farming zone, (**C**) the sewage-accepting zone, (**D**) the enclosure for a relatively concentrated fish farming, and (**E**): the agricultural runoff-accepting zone (stagnant water zone at the end region of the reservoir). The numbers (1–30) show the exact sampling location. ★: Geographic centre of Sancha reservoir and the sampling point of the sediment core. This figure was created by Binyan Jia and modified by Jen-How Huang using Arcgis10.1, Adobe Photoshop CS6 and Adobe Fireworks CS5.

The total P contents in surface sediments (0–5 cm deep) at all sites were all below 3.5 mg g^−1^ with only one exception at district B containing 11.2 mg g^−1^ P. Phosphorus surface contents showed a clear pattern with an order of B ≥ A > C and D > E (Fig. 1). Similar to the samples from the sediment profile from 42 to 27 cm depth, organic- and HCl-P were the major components, each accounting for 20–40% of total P at district B (Table S2). The importance of residual-P and NaOH-P fractions was comparably low (10–25%) and NH_4_F-fraction was almost negligible at all districts. Without considering the outlier, total P correlated significantly with MgCl_2_- (r = 0.77, p < 0.001), NaOH- (r = 0.84, p < 0.001), HCl- (r = 0.83, p < 0.001), organic (r = 0.57, p < 0.001) and residual P (r = 0.85, p < 0.001) (Figure S6). Significant correlations were also found between organic P and C (r = 0.75, p < 0.001) and between HCl-P and Ca (r = 0.54, p = 0.002, Figure S7).

### Characterisation of fish feed and water-purification reagents for fish farming

Fish feed and water-purification reagents are the most important additives of fish farming. The contents of P in four representative fish feed samples were between 13.1 and 18.1 mg g^−1^ (Table 1). Noticeably, MgCl_2_- and residual P prevailed in fish feed, accounting for 13-22% and 72–83% of total P, respectively. The water-purification reagent is applied to sustain the water quality during fish farming, which contained much less P (1.19–1.32 mg g^−1^) predominately in the form of residual P (0.70–1.01 mg g^−1^) (Table 1). The analysis of particle size distribution showed that ~50% volume of the water insoluble particle in the water-purification reagent was larger than 100 μm (Fig. 4). In the four commercial fish feed samples, two contained most of the insoluble particles larger than 100 μm and the other two included ~30–50% volume of the insoluble particles larger than 100 μm (Fig. 4). Fish feed contains much fewer minerals (11 ± 1.5%, n = 4) than water-purification reagents (80 ± 1.7%, n = 4). XRD analysis of the commercially available fish feed indicated the presence of quartz (Fig. 5). Both XRD and Raman spectroscopy revealed the presence of quartz and albite in the water-purification reagent (Fig. 5). The manufacturer claimed apatite as one of the major ingredients in the fish feed. Although IR spectroscopy clearly indentified the presence of phosphate in the fish feed and water-purification reagents based on the detection of band 460, 560–600 and 1020–1120 cm^−1^, a more precise speciation was difficult due to the interference from the other ingredients (Fig. 6). XRD and Raman spectroscopy are capable of sensitive detection of apatite^24^,^25^. Thus, the absence of apatite with our XRD (detection limit of 1%) and Raman spectroscopy implies that apatite can only be a rare accessory if present at all, reflecting well with the trace HCl-P found in fish feed (Table 1). The release of P in the fish feed into water buffered at pH 7.5 showed first a rapid release of ~1.5 mg g^−1^ P and slowed down following the 4^th^ day of lab incubation (Figure S8). After 18 days, the P released from all fish feed in the supernatant was below the detection limit. Depending on the fish feed, only 20–30% of total P in fish feed was released during the 18-day batch experiment.

### Sorption of phosphate to sediments and the water-purification reagents

The sorption isotherm of phosphate to sediments from different depths showed a higher affinity of phosphate to sediment at depths of 2 and 41 cm (K_d_ = 95.5 and 109 L kg^−1^, respectively) than at 12, 17 and 21 cm (K_d_ = 47.7, 50.7 and 61.3 L kg^−1^, respectively; see Fig. 7), which originated from the time during intensive fish farming. In comparison, the K_d_ values of phosphate sorption to water-purification reagents (7.85 and 16.3 L kg^−1^ with particle sizes larger and smaller than 63 μm, respectively) and fish feed (K_d_ = 7.68 and 8.50 L kg^−1^).

## Discussion

The detailed investigation of the sediment cores has highlighted that the P dynamic in the sediment was mainly governed by the human activities during the past 70 years. The highly significant correlation between P vertical distribution in the sediment profile and the history of P discharges from fish farming indicates fish farming as the major source of sediment P. This hypothesis is also supported by (1) the elevated sedimentation rate starting in 1990^22^ (Figure S2a) and more precisely the highly synchronised carbon mass accumulation rate with that of P (Figures S2b and S2c). Huang *et al.*^26^ evidenced that fish farming led to a significantly higher level of sedimentation and organic loadings, as well as organic matter-enriched sediments; (2) the highly significant and positive correlation between the P content in sediments and the proportion of grains > 64 μm in the sediment profile originates predominately from the fish feed and water-purification reagent (Fig. 4), probably in the form of quartz (Figs 5 and 3) the high contents of P result from high proportions of residual P in the fish feed and water-purification reagent (Table 1).

The spatial distribution of P in surface sediments clearly reflects the current hydrological conditions together with different human activities at different districts of Sancha reservoir during 2005–2010. The contents of total sediment P were lowest in district E, reflecting that its P input mostly originated from atmospheric deposition and runoff impacted by agricultural activities (Table S2). The comparably higher P accumulation in the sediment at district A and B corresponded with the high fish farming activities in this location. The slow runoff flow from the Tiaodeng and Longyun Rivers flows through district E, D, and C facilitated self-purification and biological utilisation of the water^27^. This also explains the lower contents of total sediment P at district C, D and E.

**Figure 2:**
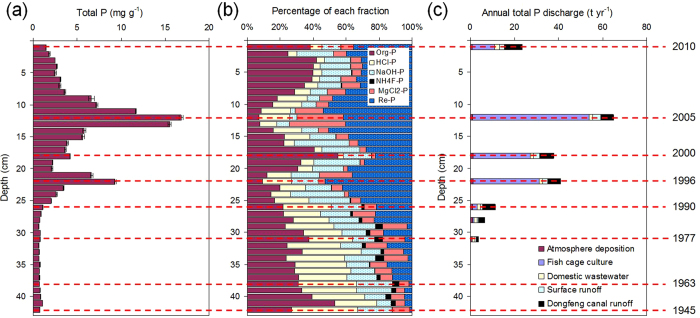
(**a**) Contents of total phosphorus and (**b**) sequential extraction based phosphorus fractionations in the sediment profile of Sancha reservoir. Org-P: organic phosphorus; HCl-P: calcium-bound phosphorus; NaOH-P: metal oxide-bound phosphorus; NH_4_F-P: aluminium-bound phosphorus; MgCl_2_-P: exchangeable phosphorus; Re-P: residual phosphorus and (**c**) Historic development of annual antrhopogenic discharge of phosphorus (in t a^−1^) and the percentage of different natural and anthropogenic inputs into Sancha reservoir, Sichuan Province, China. Data presented in Figure 2**c** comes from Jianyang Archives Bureau, Sichuan Meteorological Bureau, Sichuan Statistical Bureau, Jianyang Statistical Bureau, Jiangyang Environmental Protection Bureau, and Sichuan Environmental Protection Bureau.

Fish farming not only increased the input of P but also substantially changed the association of P with different solid phases of the lake sediment. The relatively constant P contents and fractionation in the sediments dated between 1945 and 1985 reflected the constant environmental condition in Sancha reservoir before 1985 well (Fig. 2c)^15^, but did not allow us elucidate those factors determining P fractionation in Sancha reservoir before fish farming began. In the sediments affected by fish farming (dated from 1987 to 2010), the predominance of residual P (~30–50% of total P) and the very strong correlation of residual P with total P reveals that residual P contributed to most of the increase in total P and its origin from the fish feed. Fish feed produced in China has general shortcomings of the form constancy of its particles and their ability to float, leading to their direct deposition into sediments if not taken up by fishes. Together with largely excessive amounts of fish feed and water-purification reagents furnished in un-managed manners, only 7% of phosphorus applied was effectively utilised for fish farming^28^. Results from sequential extraction (Table 1) and dissolution experiments (Figure S8) reflect the fact that most P in the fish feed and water-purification reagent that is not taken up by fishes deposited into surface sediments predominately as residual P. Thus, only a small part of it dissolved in the water and eventually adsorbed to suspended and sedimented particles. Further analysis at the molecular scale is required to point out the chemical form of P associated with the residual phases to elucidate its potential long-term influence on P biogeochemical cycling in the lake environment.

**Figure 3:**
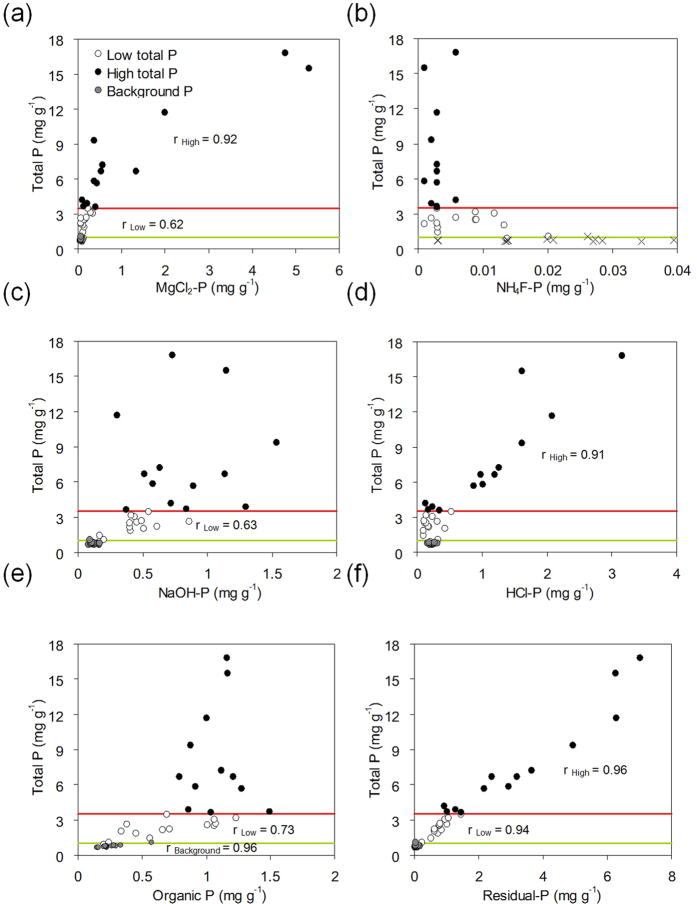
Correlations between the contents of total P and different sequential extraction defined P fractions ((**a**): MgCl_2_, (**b**): NH_4_F, (**c**): NaOH, (**d**) HCl, (**e**) orgnaic and (**f**) residual P) in the sediment profile of Sancha reservoir. Threshold contents of total P: 3.5 mg kg^−1^ (red lines) and 1 mg g^−1^ (green lines). Linear correlation coefficient values of significant correlations are shown.

**Figure 4:**
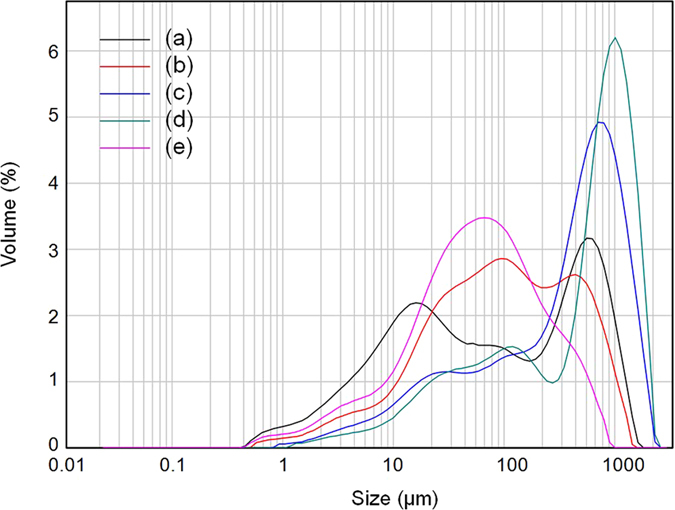
Distribution of particle size in the (**a**) water-purification reagent and (**b–e**) fish feed.

**Figure 5:**
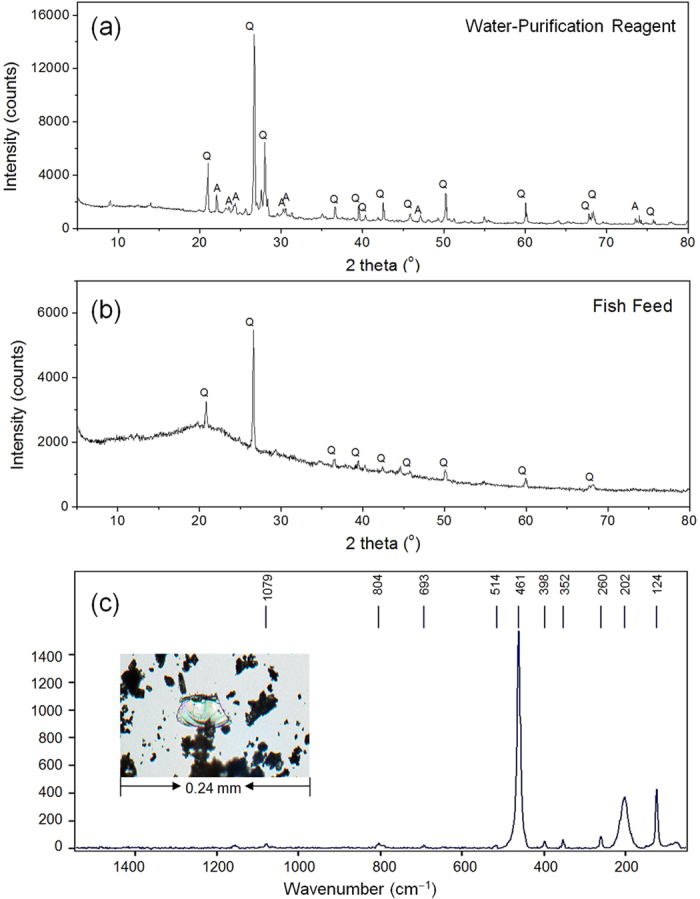
(**a**) XRD spectrum of (**a**) water-purification reagents and (**b**) fish feed and (**c**) Raman spectrum of quartz identified in the clean-up reagent. Q: quartz; A: albite.

**Figure 6:**
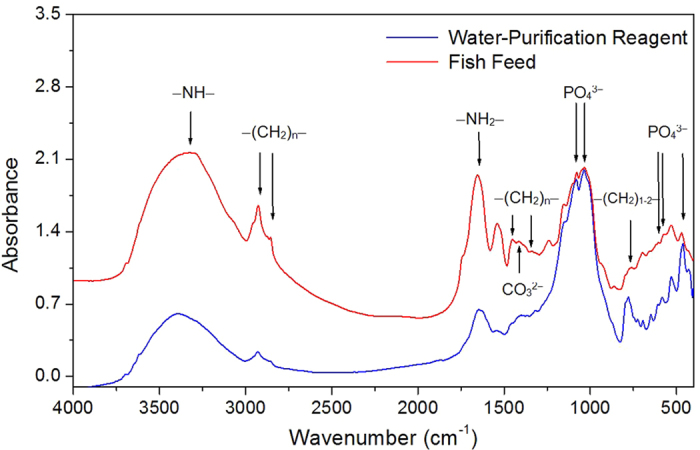
IR spectra of fish feed and water purification reagents.

Intensified fish farming largely increased P input in the form of HCl-P in the sediments, as indicated by the strong correlation between HCl-P and both total P and Ca, only at high P levels (Fig. 3d and S5). Hydrochloride-P is mainly derived from biological debris, calcium carbonate combined with P, native detrital apatite and other inorganic P sources^4^,^11^,^29^. Fish farming has been evidenced to enhance the accumulation of Ca-bound P in the sediment and fish feed and faeces were suggested as its origins^19^,^20^. Considering the fact that HCl-P was trace in fish feed and water-purification reagents (0.03–0.72 mg g^−1^, Table 1), most increases of HCl-P in the sediments under intensive fish farming (0.18–3.2 mg g^−1^, Fig. 3d) had different origins e.g., biological debris induced by increased nutrient inputs or fish faeces. Further research is needed to clarify these points. Organic substances play an important role in the P storage in the near natural sediments, as indicated by the significant correlations between organic P; both total P and C only found at low P levels and under natural conditions (Fig. 3e, S5a, S6e and S7a). This emphasises the higher relevance of P-containing macromolecules synthesised by aquatic organisms at low P levels which enter the organic P pool upon the death of the organism^30^. Residual P was immobile and HCl-P was relatively stable only sensitive to low pH^31^,^32^. Mobilising organic and NaOH-P acquired usually a remarkable change of environmental conditions e.g., pH and redox potential, which accelerate microbial decomposition of organic matter and weaken P sorption affinity, respectively^29^,^33^. However, these conditions are mostly stable in Sancha reservoir (e.g. pH ~8 since 1989), with the exception of certain environmental events, e.g. the anoxia leading to a mass death of fishes. Thus, the shifts of P fractionation among organic, residual, NaOH- and HCl-P in sediments under different fish farming intensities generally changed the mobility of P little.

**Figure 7:**
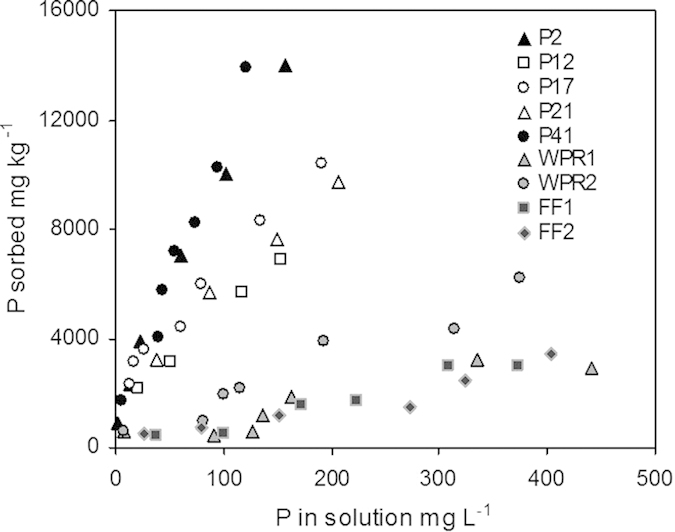
Sorption isotherms of phosphate to sediment materials from selected layers of the sediment profiles (P2 (2 cm depth), P12 (12 cm depth), P17 (17 cm depth), P21 (21 cm depth), and P41 (41 cm depth)), to water-purification reagent with particle size larger than 63 μm (WPR1) and smaller than 63 μm (WPR2) and to fish feed (FF1 and FF2).

Magnesium chloride extracts easily mobilisable P but may not exchange strongly adsorbed P from the sediment^34^, whereas NaOH extraction released P by elevating pH during extraction and subsequently decreased P sorption affinity to metal (hydr)oxide phases in sediments^35^,^36^. Thus, NaOH-P may represent the strongly adsorbed P in the sediment e.g. via innersphere complexation, which may be hardly released by MgCl_2_^35^,^37^. The natural sediments in Sancha reservoir are mainly composed of minerals with clay- and silt-sized particles^22^. Large amounts of sand-sized minerals from the water-purification reagent and fish feed deposited into sediments acted as additional P sink despite the lower P sorption affinity (Fig. 7)^38^. Thus, sand-sized minerals became the major sorbents of P, explaining the larger increase of MgCl_2_-P contents with total P at high rather than low levels (Fig. 3a), and consequently increased the pools of easily mobilisable P in the sediments with elevated activities of fish farming.

In the surface sediments, the dependence of total P on P associated with different fractions agreed well with those in the sediment profile that was slightly affected by fish farming (Figure S6). One surface sediment containing 11.2 mg kg^−1^ P (see square in Figures S6 and S7) behaved similarly to the sediments at high P levels. Thus, the results from the surface sediments support the aforementioned hypothesis conclusively. Our findings point out the mechanism responsible for decreasing P sorption affinity due to fish farming in reservoir sediments. Sand-sized minerals as additional sorbents increase the pool of easily mobilisable phosphorus in sediments, which will slow down the recovery of reservoir water due to its rapid re-mobilisation.

## Methods

### Site description

Sancha reservoir is located in Jianyang Municipality in southwestern Sichuan Province, southwest China (Fig. 1). It is the second largest reservoir in Sichuan Province, with water from the discharge of the Dujiangyan irrigation network (Minjiang River). Sancha reservoir has an area of 27 km^2^ and its depth averages 8.3 m. In 1977, a dam was built to introduce Jiangxi River water into Sancha reservoir. The reservoir is the major water supply for Jianyang Municipality with highest water level (460 m a.s.l.) in March-June and lowest in July-August (456 m a.s.l.). Based on the local socio-economic status and its function for water resource and pollution regulation, the total reservoir area can be divided into five zones: (A) the main reservoir inflow (south main canal) zone, (B) the highly concentrated fish farming zone, (C) the sewage-accepting zone, (D) the enclosure for a relatively concentrated fish farming, and (E) the agricultural runoff-accepting zone (stagnant water zone at the end region of the reservoir; see Fig. 1). The major water inflow into Sancha reservoir is from the southern watershed of northern Dujiangyan irrigation network, predominantly between July and September (80% of the water volume); the remainder is derived from rainfall and 2 creeks in the southwest (Tiaodeng and Longyun Rivers). The only reservoir outlet is in the Northeast of the reservoir. It either enters into Tuojiang River or an irrigation canal, which was controlled manually (Fig. 1). Because the inlet and outlet of Sancha reservoir are close in distance (~2.5 km), much of the runoff from the south canal of Dujiangyan irrigation network only passes through district A and B and then directly discharges into the Tuojiang River, a tributary of the Yangtze River. The average depth of the reservoir in this region is greater than 15 m. The water has a rapid flow and is more turbulent than in the other reservoir districts. The runoff originated from the Tiaodeng and Longyun Rivers into Sancha reservoir discharges into the Tuojiang River, flowing though district C, D and E. The average depth of reservoir in these zones is less than 10 metres, and the flow is comparably slow and primarily stratified.

### Sediment sampling

Surface sediments were sampled in July 2010 at district A, B, C, D and E, with each district being comprised of 4–8 sampling locations (numbered 1–30, Fig. 1). Duplicate sediment samples (0–5 cm deep) were collected with a self-gravity columnar bottom sampler (ZH7690, Nanjing, China) at each sampling location. In parallel, a sediment core with a length of 42 cm was sampled at the geographic centre of Sancha reservoir, (marked with ★ in Fig. 1), at the deepest point of Sancha reservoir, with the highest probability of sampling a continuous sediment core. Since deep water facilitates the preservation of sediments, continuous layers of sediments can most probably be accessed at such places of the reservoir. The sampling point is located on the previous riverbed of Jiangxi River, on which the dam was built in 1977 and subsequently Sancha reservoir formed. Thus, the deposition of sediment material is continuous here. The sediment core was immediately sliced into 1 cm thick pieces *in situ*, sealed individually in polyethylene bags, and ice cooled for transport to the laboratory. All sediment samples were sieved to 200 μm, freeze-dried (Eppendorf 5804R, A-4-44, Germany) and ground for further analysis.

### Phosphorus sequential extraction in sediment samples

Sequential extraction was performed in triplicate with 0.2–0.3 g sediment samples in 50 mL centrifuge tubes using a method modified from Ruttenberg^39^ and Zhang *et al.*^40^. Thus, the P pools in sediments were fractionised into (1) exchangeable fraction (MgCl_2_-P): 30 mL 1 M MgCl_2_ at 25 °C, 16 h, (2) aluminium-bound fraction (NH_4_F-P): 30 mL 0.5 M NH_4_F at pH 8.2 and 25 °C, 16 h, (3) iron (hydr)oxide-bound fraction (NaOH-P): 30 mL 0.1 M NaOH and 0.5 M Na_2_CO_3_ at 25 °C, 16 h, and (4) calcium and carbonate-bound P (HCl-P): 30 mL 1 M HCl at 25 °C, 16 h. For the determination of organic P, other 0.2–0.3 g sediment samples were taken for extraction with 30 mL 1 M HCl at 25 °C for 16 h. Thereafter, the residue was heated to 450 °C in a muffle furnace (CWF 12/13, Carbolite^®^, United Kindom) for 4 h to mineralise organic P (as organic P). For the analysis of total P, sediment samples (0.25 g) were heated with a mixture of concentrated H_2_SO_4_ and HClO_4_ (6:1 (v/v)) until completely dissolved. Residual P was calculated by subtracting the sum of organic-, MgCl_2_, NH_4_F-, NaOH- and HCl-P from total P. Generally, after each step of extraction, the sample was centrifuged for 30 min at 4500 *g*. All resulting supernatants were filtered to 0.45 μm (AL-01, FB-10T, China) and analysed for P with spectrophotometer (PhotoLab^®^ 6100 VIS, WTW, Weilheim, Germany).

### Characterisation of fish feed and water-purification reagents

The same fish feed and water-purifications reagents previously used for fish farming in Sancha reservoir were purchased in local markets in SW-China. The mineral composition in fish feed and water-purification reagents was verified mutually with IR, XRD and Raman spectroscopy. For Raman spectroscopic analysis, particles of fish feed and water purification reagents were mounted on a microscope slide using Hillquist raisin. Afterwards, they were covered with Laromin araldite and polished to a thickness of 25 μm. Raman analyses were performed in the central section of the polished grains to avoid fluorescence using a Bruker Senterra dispersive microscope spectrometer (Bruker, Kalsruhe, Germany) equipped with a green laser (532 nm) with a power of 20 mW. Measurements were performed using objective lenses with a fifty-fold magnification and an aperture of 50 μm. Mineral identification was performed using the RRUFF database^41^. For XRD analysis, all samples were first ground with an agate mortar and then placed into the plastic sample holder for analysis with an X’Pert Pro XRD (PANalytical, Almelo, the Netherlands). Minerals were identified according to the ICCD database. Another portion of ground fish feed and water-purification reagents were mixed with KBr at ratios of 1:100–1:200 to be pelleted for the subsequent analysis with FT-IR spectroscopy (VEXT 70, Bruker, Germany).

### Batch sorption and desorption experiments - binary phosphate sorption experiments

Binary phosphate sorption experiments were performed in suspensions of sediments and the water-purification reagent, which was washed with deionised water until a constant weight, in PIPES-buffered solutions (10 mM, pH 7), were allowed to equilibrate while stirring for 24 h, and were then spiked in a step-wise fashion with phosphate to achieve final contents in the range 20−500 mg L^−1^. After each step, a 20 mL subsample was removed from the main vessel under vigorous stirring and pipetted into a 50 mL glass bottle, which was placed on an over-head shaker for equilibration. All phosphate-spiked suspensions were equilibrated at room temperature for 24 h. Following equilibration, the solutions were syringe-filtered through 0.2 μm nitrocellulose filter membranes, and analysed for phosphate using spectrophotometric determination based on molybdenum blue chemistry. The adsorbed amounts of phosphate were calculated from the difference between the initial and final phosphate concentrations.

### Fish feed dissolution experiment

Approximately 0.5 g of fish feed was mixed with 15 mL de-ionised water buffered with 1 mM PIPES at pH 7.5 in a 50 mL polyethylene tube and was equilibrated on an end-over-end shaker (50 rpm). The experiment lasted for 18 days and the solution was sampled with an interval of 1-3 days at the first half and of 4-5 days at the second half of the experiment. For each sampling, the supernatant was decanted and centrifuged at 2000*g* for 20 minutes at room temperature. The supernatants were syringe-filtered through 0.2 μm nitrocellulose filter membranes, and analysed for phosphate using spectrophotometric determination based on molybdenum blue chemistry. Thereafter, 15 mL new 1 mM PIPES solution was added for further dissolution experiments.

### Grain size analysis and sediment core dating

Grain size analysis was completed with laser particle analyser (Mastersize2000, Malvern, UK). Dating sediment core was achieved by measuring ^137^Cs and ^210^Pb using a high-purity germanium γ spectrum analysis system (EG&G Ortec, USA). More information was described in Supplementary Information and has been published in Jia *et al.*^22^.

## Additional Information

**How to cite this article**: Jia, B. *et al.* Impact of Fish Farming on Phosphorus in Reservoir Sediments. *Sci. Rep.* **5**, 16617; doi: 10.1038/srep16617 (2015).

## Supplementary Material

Supplementary Information

## Figures and Tables

**Table 1:** Contents of total phosphorus and phosphorus in different fractions based on sequential extraction for sediments in commercial available fish feed and water-purification reagents (mg g^−1^).

	Total phosphorus(mg g^−1^)	Organic phosphorus(mg g^−1^)	Inorganic phosphorus (mg g^−1^)				
			MgCl_2_-P	NH_4_F-P	NaOH-P	HCl-P	Residual-P
*Fish feed*							
1	18.1 ± 0.14	0.14 ± 0.01	4.00 ± 0.06	0.02 ± 0.01	0.15 ± 0.03	0.63 ± 0.11	13.3 ± 0.25
2	14.5 ± 0.13	0.07 ± 0.01	1.87 ± 0.15	0.12 ± 0.02	0.03 ± 0.01	0.06 ± 0.02	12.4 ± 0.23
3	15.3 ± 0.34	0.18 ± 0.01	2.95 ± 0.29	0.24 ± 0.05	0.33 ± 0.02	0.72 ± 0.07	11.1 ± 0.58
4	13.1 ± 0.24	0.14 ± 0.01	2.63 ± 0.21	0.11 ± 0.01	0.06 ± 0.01	0.12 ± 0.02	10.2 ± 0.40
*Water-purification reagent*							
<63 μm	1.32 ± 0.04	0.24 ± 0.01	0.19 ± 0.02	0.07 ± 0.01	0.01 ± 0.01	0.04 ± 0.02	1.01 ± 0.06
>63 μm	1.19 ± 0.06	0.27 ± 0.01	0.32 ± 0.02	0.05 ± 0.01	0.09 ± 0.01	0.03 ± 0.01	0.70 ± 0.02

Mean values and standard errors of three extraction and digestion replicates for the fish feed and water-purification reagent are shown. −: below detection limit
