# Supplementary material for: Impact of Fish Farming on Phosphorus in Reservoir Sediments
Source: Sci Rep. 2015 Nov 18;5:16617. doi: 10.1038/srep16617 (PMC4649609; doi:10.1038/srep16617)
Supplement: Supplementary Information [file srep16617-s1.doc]

**Supplementary material**

***Scientific Reports***

Impact of Fish Farming on Phosphorus in Reservoir Sediments

Binyang Jia^a, b^, Ya Tang^a^, Liyan Tian^c^, Leander Franz^d^, Christine Alewell^c^, Jen-How Huang^c^, *

*^a^ College of Architecture and Environment, Sichuan University, Chengdu 610065, People’s Republic China.*

*^b^ Chengdu Academy of Environmental Sciences, Chengdu 610065, People’s Republic China.*

*^c^ Environmental Geosciences, University of Basel, CH-4056 Basel, Switzerland*

*^d^ Mineralogy and Petrography, University of Basel, CH-4056 Basel, Switzerland*

**11 pages, 2 Tables and 8 Figures**

**Characterisation of the current aqueous environment in Sancha reservoir**

The investigation at different districts of Sancha reservoir in 2010 showed a moderate eutrophication, with pH of 7.3-8.5, a transparency of 117 cm and an averaged total organic carbon concentration of 2.39 mg L^−1^ (Table S1). Dissolved oxygen in the surface lake water was 4.6-14 mg L^−1^ and the averaged COD_Cr_ and BOD_5_ were 17.3 and 4.1 mg L^−1^ (Table S1). The concentrations of N and P averaged 1.57 mg L^−1^ and 68 µg L^−1^, respectively, with small variations at different sites. The total P concentrations were higher in the bottom water with concentrations up to 140 µg L^−1^, however, without statistical significance due to the high heterogeneity of the bottom water. In comparison, the concentrations of total P in sediment pore water (257 to 622 µg L^−1^) were remarkably higher than those in the surface water lake and increased with the sediment depth.

**Table S1.** Diaphaneity, concentrations of total nitrogen (total N), total phosphorus (total P) and surface layer dissolved oxygen, chemical (COD_Mn_), biological oxygen demand (BOD) and status of eutrophication in the water environment in Sancha reservoir from 1978 to 2010*

| Parameters | 1978-1985 | 1986-1990 | 1991-2000 | 2000-2005 | 2005-2010 |
| --- | --- | --- | --- | --- | --- |
| Diaphaneity (m) | >10 | 1.2-2.8 | 0.8-1 | 0.7-1 | 0.5-1.5 |
| Total N (mg L^−1^) | <0.5 | 0.5-1.0 | 0.5-1.5 | 1.0-1.5 | 1.0-2.0 |
| Total P (mg L^−1^) | <0.025 | 0.025-0.05 | 0.05-0.15 | 0.15-0.10 | 0.15-0.10 |
| Surface layer dissolved oxygen (mg L^−1^) | >6 | >6 | 6-4 | 6-4 | >5 |
| COD_Mn_（mg L^−1^) | <3 | 4-6 | 4-10 | 4-6 | 4-6 |
| BOD_5_ (mg L^−1^) | <2 | <4 | 4-8 | 4-6 | 4 |
| Status of eutrophication | oligotrophic | mesotrophic | eutrophic | eutrophic | eutrophic |

*: Data adapted from the Archives Bureau, Statistical Bureau and Environmental Protection Bureau of Jianyang City, Sichuan Province, China and the Meteorological Bureau, Statistical Bureau and Environmental Protection Bureau of Sichuan Province, China.

**Table S2**. Contents of total phosphorus in sediments (mg g^−1^) and averaged percentages (%) of different phosphorous fractions based on sequential extraction in surface sediments (0-5 cm) at different districts of Sancha reservoir. Mean values and standard errors of 4-8 spatial replicates for sediments are shown.

| District | Total phosphorus (mg g^−1^) | Organic phosphorus (%) | Inorganic phosphorus (%) | | | | |
| --- | --- | --- | --- | --- | --- | --- | --- |
|  | Median, Min - Max |  | MgCl_2_-P | NH_4_F-P | NaOH-P | HCl-P | Residual-P |
| *Sediment* | |  |  |  |  |  |  |
| A | 1.87, 1.75 - 3.56 | 24.5 ± 3.84 | 9.98 ± 2.89 | 1.16 ± 0.27 | 12.7 ± 3.55 | 25.9 ± 3.65 | 25.8 ± 8.60 |
| B | 2.30, 1.68 - 11.2 | 26.7 ± 4.57 | 6.55 ± 0.64 | 1.04 ± 0.17 | 16.5 ± 2.20 | 29.3 ± 4.30 | 20.0 ± 2.59 |
| C | 1.21, 0.86 - 1.84 | 32.4 ± 5.61 | 6.18 ± 1.52 | 1.67 ± 0.38 | 12.1 ± 1.29 | 32.9 ± 0.79 | 14.8 ± 3.41 |
| D | 1.27, 1.07 - 2.24 | 41.5 ± 4.77 | 5.75 ± 0.93 | 2.50 ± 0.52 | 12.7 ± 1.54 | 23.7 ± 3.42 | 13.8 ± 3.74 |
| E | 0.68, 0.36 - 1.04 | 30.9 ± 6.41 | 6.49 ± 1.51 | 5.45 ± 2.24 | 14.6 ± 2.29 | 32.0 ± 4.49 | 10.5 ± 2.96 |
| All Area | 1.71, 0.36 - 11.2 | 30.9 ± 2.33 | 6.76 ± 0.61 | 2.37 ± 0.53 | 14.1 ± 0.93 | 29.6 ± 1.66 | 16.2 ± 1.92 |

A, B, C, D, and E represent the sampling districts. A: the main reservoir inflow (south main canal) zone, B: the highly concentrated fish farming zone, C: the sewage-accepting zone, D: the enclosure for a relatively concentrated fish farming, and E: the agricultural runoff-accepting zone (stagnant water zone at the end region of the reservoir).

**Determination of radionuclides and sediment core dating.** Dating sediment core was performed at the State Key Laboratory of Lake Science and Environments, Nanjing Institute of Geography and Limnology, Chinese Academy of Sciences. The chronosequence of the sediments in the Sancha reservoir was established based on the calculated results of ^210^Pb , combining the ^137^Cs dating results and the constant rate of supply (CRS) (Figure S1). ^137^Cs and ^210^Pb were measured using a high-purity germanium γ spectrum analysis system (EG&G Ortec, USA). The standard source and activity calibration were provided by the China Institute of Atomic Energy. The sample was sealed in a vial for 3 weeks to reach radioactive equilibrium, and the specific activities of ^210^Pb and ^137^Cs were then measured with 46.5 and 661.6 keV γ spectra, respectively, with a measurement error of less than 5%. The ^210^Pb_exc_ profile showed a continuous increase but no exponential distribution (Figure S1b) indicating that the CIC model is not suitable for sediment dating at Sancha reservoir . Therefore, the CRS model was used to calculate the geochronology of our sediment core taking into account the age of the surface sediment deposited in 2002. The results of the SCP profile confirmed that the CRS model of ^210^Pb is suitable to establish the time sequence . Based on this geochronology the mass accumulation rate (MAR) was calculated. More details have been published in Jia et al. .

(b)

(a)


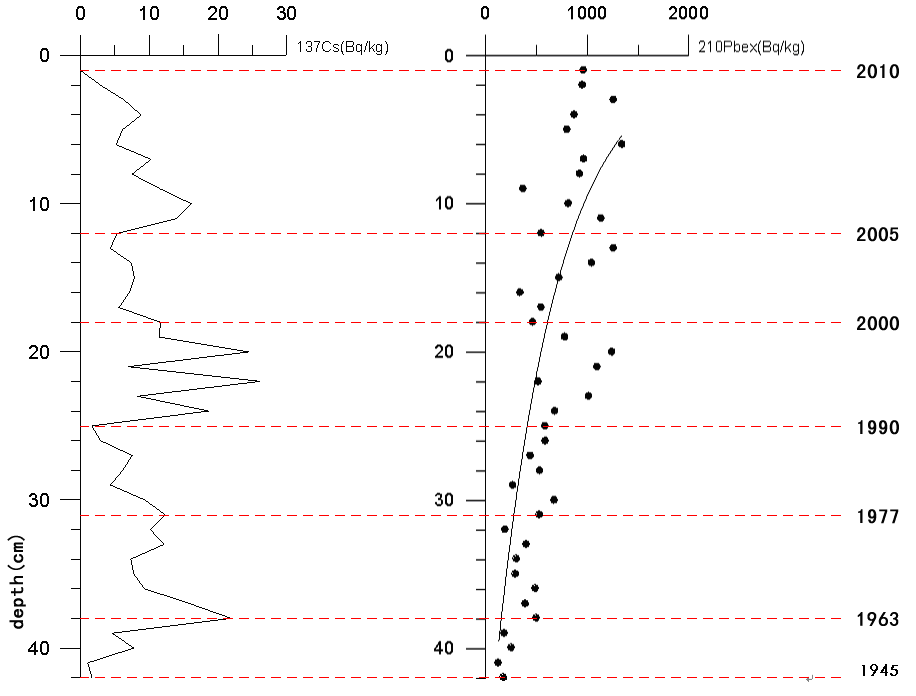


Figure S1│ Distribution of (a) ^137^Cs and (b) ^210^Pb_ex_ in the in the sediment profile of Sancha reservoir.

**Sedimentation and mass accumulation rate.** The sedimentation rate of Sancha reservoir was 0.24 - 4.67 cm a^−1^, and the carbon and phosphorus mass accumulation rates were 0.56 - 8.66 g cm^−2^·yr^−1^ and 0.16 - 4.88 mg cm^−2^·yr^−1^ Since the construction of the reservoir in 1977, the sedimentation rate has been increasing, peaking in 2009 (4.67 cm yr^−1^) (Figure S2). Both carbon and phosphorus mass accumulation rates peaked in 2005 and 1996.

(c)

(a)

(b)

1963

1977

1996

2000

2005

2010

1990

Figure. S2│ (a) Sedimentation rate and (b) carbon mass accumulation rate of Sancha reservoir sediments

**Grain size analysis and granulometric composition.** Sample analysis was completed at the Key Laboratory for Lake Sedimentation and Environment of the Nanjing Institute of Geography & Limnology, CAS. A Mastersize2000 Laser Particle Analyzer manufactured by MALVERN of UK was used. The parallel analysis error of each fraction was less than 5%.

Before 1985, the sediments mainly consisted of silt- and clay-sized particles (below 27 cm) (Figure S3). A change happened during 1985 to 2000 (26-18 cm), with silt- and clay-sized particles decreasing and sand-sized particles increasing; the content of sand-sized particles reached a maximum in 1988 (at 22 cm), with 44.9% of the particles larger than 64 µm and 12.7% between 32 and 64 µm; later, the amount of silt- and clay-sized particles gradually increased. Another change occurred during 2000 to 2009 (18-2 cm), with silt- and clay-sized particles decreasing and sand-sized particles increasing; the content of sand-level particles reached a maximum in 2005 (at 12 cm), with particles larger than 64 µm constituting 69.3% of the sediment (Figure S3).


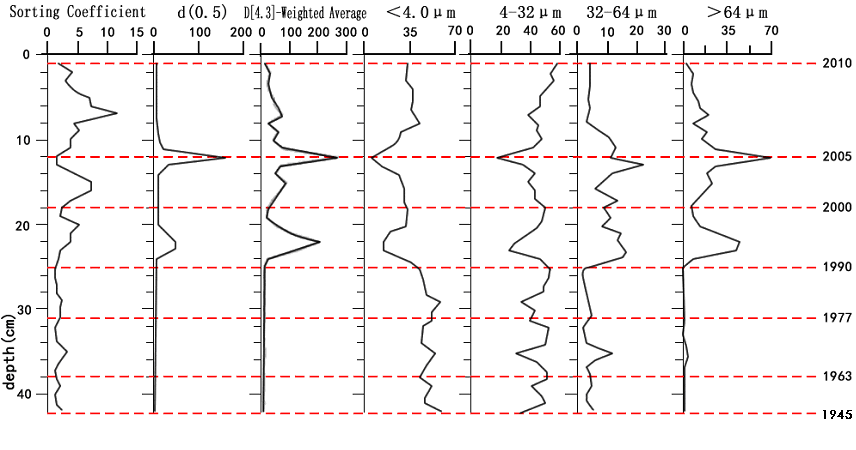


Figure S3│ Size distribution of sediment grain in the sediment profile of Sancha reservoir.

**Collection of other relevant data.** Information regarding social economy, meteorology, water quality and pollution sources were collected from the Jianyang Archives Bureau, the Sichuan Meteorological Bureau, the Sichuan Statistical Bureau, the Jianyang Statistical Bureau, the Jiangyang Environmental Protection Bureau, and the Sichuan Environmental Protection Bureau.

r = 0.84

r = -0.86

(a)

(b)

Figure S4│ Correlations between total P concentrations and the percentage of grains (a) larger than 64 µm and (b) smaller than 32 µm in the sediment profile of Sancha reservoir. Linear correlation coefficients are shown.

(b)

(a)

r _Low_ = 0.91

r _High_ = 0.83

Figure S5│ Correlations between the concentrations of (a) organic P with C and (b) HCl-P with Ca in the sediment profile of Sancha reservoir. Threshold concentrations of total P: 3.5 mg kg^−1^ (between low and high total P) and 1 mg kg^−1^ (between low total and background P). Linear correlation coefficients of significant correlations are shown.

| (a)  (c)  (d)  (b)  r = 0.77 |  |
| --- | --- |
| (f)  (e)  r = 0.84 | r = 0.83 |
| r = 0.57 | r = 0.85 |

Figure S6│ Correlations between the concentrations of total P and total C, Ca and different sequential extraction defined P fractions ((a): C, (b): Ca, (c): NaOH-P, (d) HCl-P, (e) organic P) in the surface sediments of Sancha reservoir. Threshold concentrations of total P: 3.5 mg kg^−1^. Linear correlation coefficients of significant correlations are shown.

(b)

(a)

r = 0.54

r = 0.75

Figure S7│ Correlations between the contents of (a) organic P with C and (b) HCl-P with Ca in the surface sediments of Sancha reservoir. Threshold contents of total P: 3.5 mg kg^−1^. Linear correlation coefficients of significant correlations are shown.

Figure S8│ Dynamics of phosphate release from different fish feeds in water buffered with 1 mM PIPES at pH 7.5. Mean values and standard errors of three replicates are shown.

**References**

Appleby, P. G. and F. Oldfield (1978). "The calculation of lead-210 dates assuming a constant rate of supply of unsupported 210Pb to the sediment." Catena **5**: 1-8.

Appleby, P. G., N. Richardson, et al. (1992). "Self-absorption corrections for well-type germanium detectors." Nuclear Instruments & Methods in Physics Research Section B-Beam Interactions with Materials and Atoms **71**(2): 228-233.

Jia, B.-Y., Y. Tang, et al. (2012). "Driving effect of human activity on the environmental change of the Sancha Lake." 2012 International Conference on Biomedical Engineering and Biotechnology (iCBEB): 1361-1366.

Wu, Y. H., S. M. Wang, et al. (2005). "Dating recent lake sediments using spheroidal carbonaceous particle (SCP)." Chinese Science Bulletin **50**(10): 1016-1020.
